# Rupture of the Ulnar Artery in a Case of Neurofibromatosis Type 1

**DOI:** 10.7759/cureus.82596

**Published:** 2025-04-19

**Authors:** Takaaki Nakano, Toshitaka Ito

**Affiliations:** 1 Department of Emergency Medicine, Shin-Yurigaoka General Hospital, Kanagawa, JPN

**Keywords:** endovascular treatment, neurofibromatosis type 1, surgical procedure, ulnar artery rupture, vascular fragility

## Abstract

Neurofibromatosis type 1 (NF1) is a genetic disorder involving an abnormality on chromosome 17, resulting in the production of the protein neurofibromin. Neurofibromin inhibits cell proliferation, and abnormalities in its encoding gene are hypothesized to trigger signals for proliferation, resulting in various lesions. Vascular fragility is a rare complication of NF1; however, ruptures of various vessels have also been reported. Here, we present a case of ulnar artery rupture treated endovascularly by puncturing the ipsilateral brachial artery, achieving excellent results.

## Introduction

Vascular fragility, a rare complication of neurofibromatosis type 1 (NF1), has an incidence ranging from 2.3% to 3.6% [1,2] and spreads to nearby vessels, both arteries and veins. [3] The major and minor complication rates of endovascular treatment (EVT) were 15% and 6%, and challenging cases have been reported. Vascular fragility is difficult to determine visually; thus, whether the surgical procedure or EVT is safer remains unknown. [4] Blood vessel vulnerability should be considered when performing surgery or invasive procedures in the vicinity of large blood vessels. Even with EVT, avoiding procedures around large blood vessels is recommended. In this case, the patient was punctured from a proximal vessel for a ruptured artery in the extremity with good results.

## Case presentation

A 47-year-old woman with no medical history was transferred to our orthopedic surgery department on day X with a diagnosis of left forearm compartment syndrome from another hospital. Surgery was performed to remove the hematoma. However, the source of the bleeding could not be identified surgically. She was discharged on day X+4 without any postoperative complications.

On day X+6, she returned to our emergency room due to sudden swelling and severe pain at the postoperative wound site.

Physical examination revealed swelling and tightness in the left forearm. She had café-au-lait spots all over her body but had never been diagnosed with neurofibromatosis type 1 (NF1). Her sister, who accompanied her, had no café-au-lait spots.

**Figure 1:**
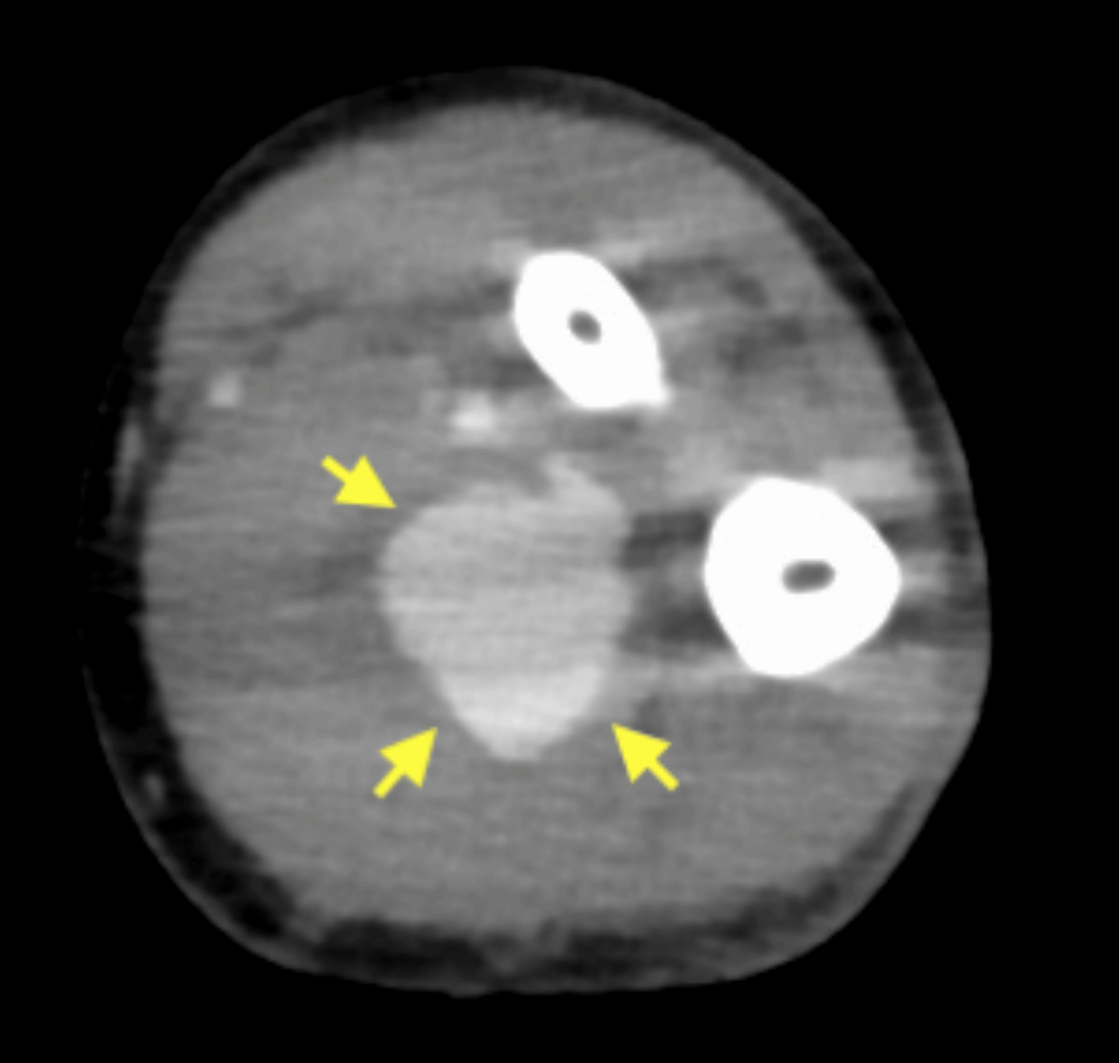
Contrast-enhanced computed tomography of the left forearm Contrast-enhanced computed tomography (CT) showing extravasation (yellow arrow) from the ulnar artery.

Contrast-enhanced computed tomography revealed a spontaneous rupture of the left ulnar artery (Figure 1). Ligation of the ulnar artery was considered; however, the ruptured artery was deep, and the surrounding vessels were vulnerable. Also, the pulsation of the radial artery was well palpable. Therefore, we decided to perform transcatheter arterial embolization (TAE).

Considering the fragility of other arteries, we planned to insert a catheter through the brachial and adjacent arteries. (At this time, the patient had a bone deformity - a symptom of NF1-causing internal rotation of the upper arm to the forearm, which complicated the puncture procedure.)

**Figure 2:**
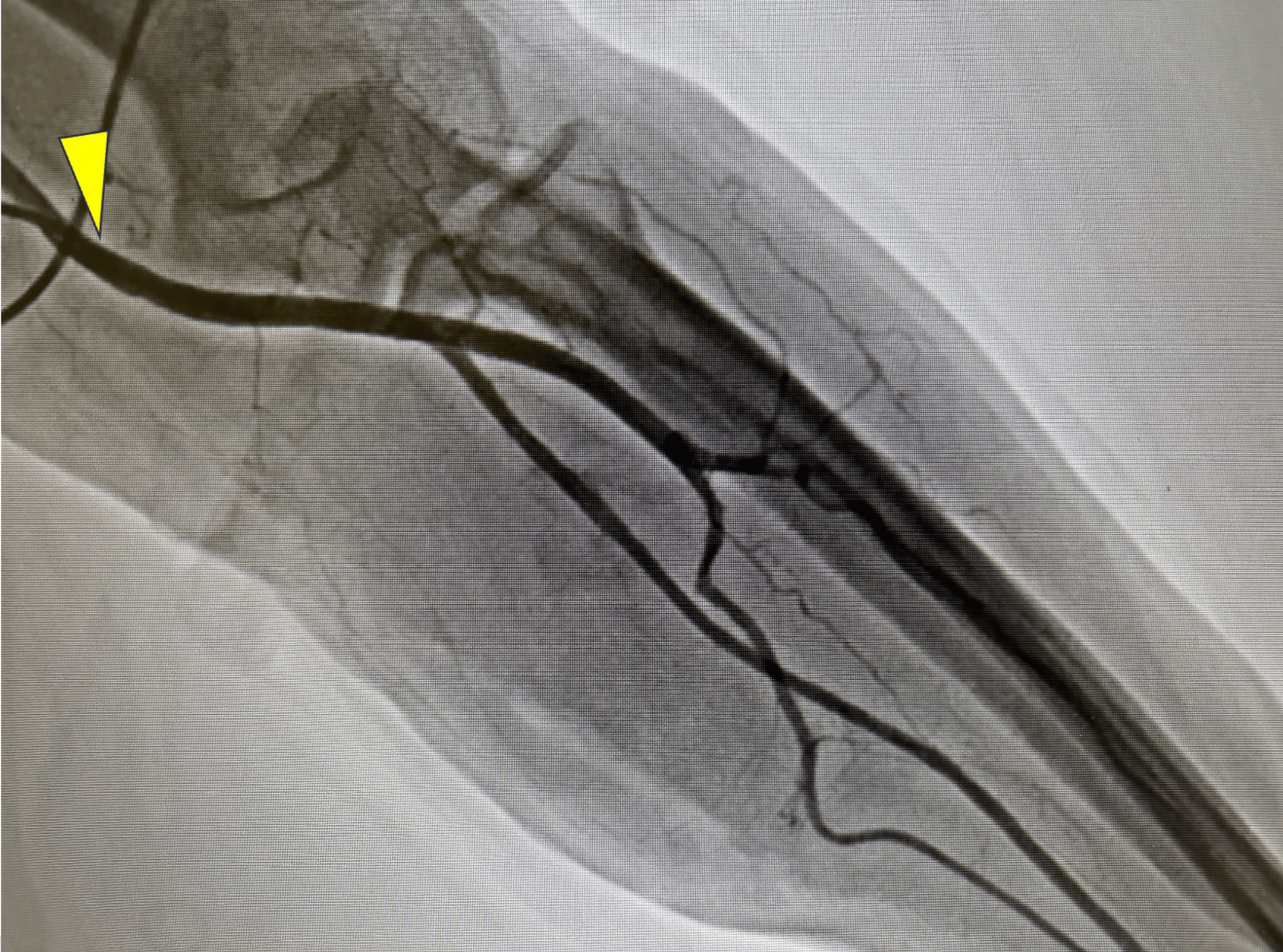
Initial angiography 4Fr Radifocus Introducer ⅡH® insertion into the brachial artery (yellow arrow). The ulnar and radial arteries are contrasted without vascular injury on initial examination through the introducer.

**Figure 3:**
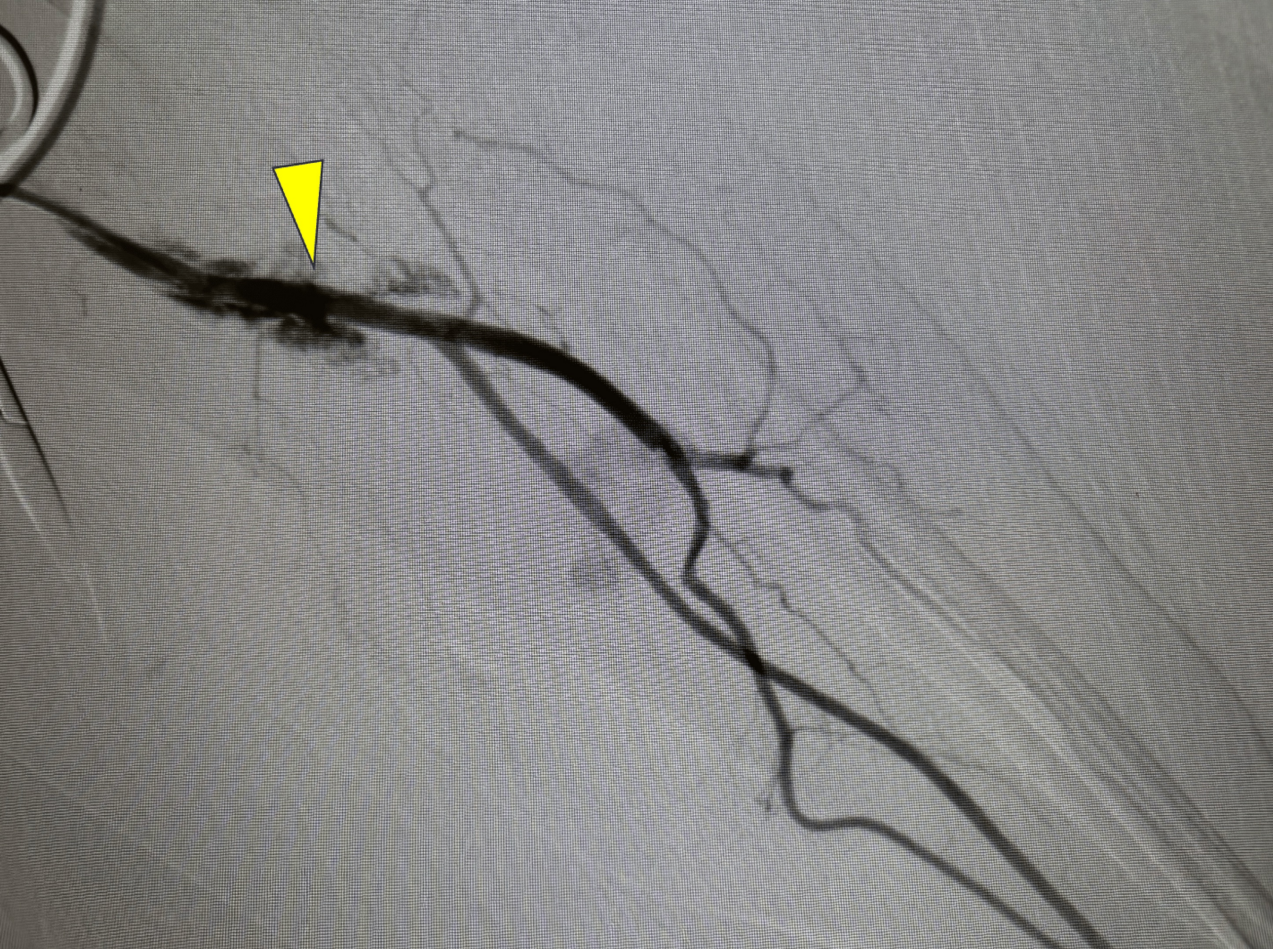
Extravasation of the contrast medium The microcatheter was inserted to obtain detailed images of the injured area in the ulnar artery. The contrast scan shows extravasation (yellow arrow) near the introducer insertion site. The procedure was continued as the vascular injury did not interfere with the endovascular treatment procedure.

**Figure 4:**
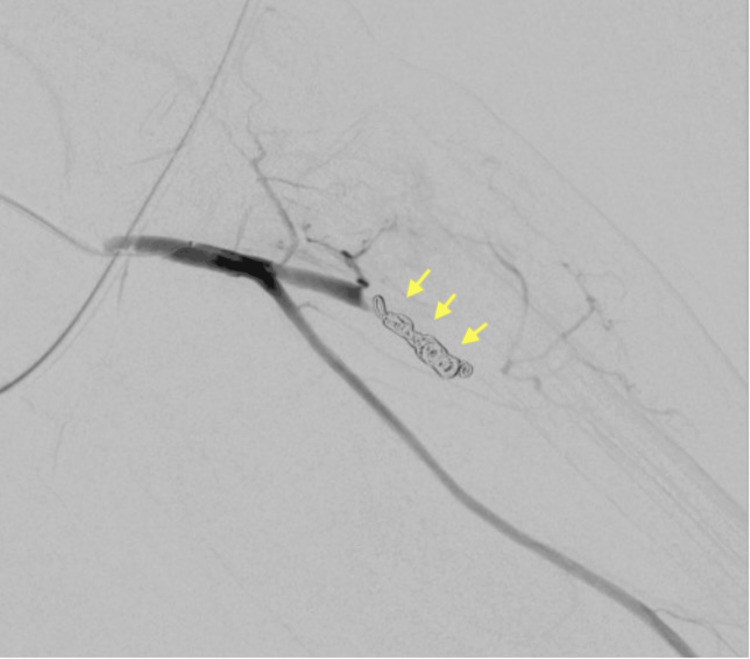
Coil embolization Coil embolization (yellow arrow) was completed without further complications (6 coils were used: Three 5 mm×2 mm Tornade®︎Embolization Microcoil and three 4 mm×2 mm Tornade®︎Embolization Microcoil). The observed extravasation near the introducer insertion site had disappeared at the last imaging. Compression of the puncture site at the time of introducer removal was performed for 20 minutes, and no postoperative puncture site swelling was observed.

A 4Fr sheath (Radifocus Introducer ⅡH®︎) was inserted into the brachial artery (Figure 2), and a microcatheter (GOLD CREST®︎ Neo) was inserted into the ulnar artery. During the insertion of the microcatheter, the vessel was injured (Figure 3), and extravasation of the contrast medium was observed. Fortunately, the leakage was manageable, and coil embolization was performed in the proximal part of the ruptured vessel (Figure 4). Finally, a radial artery scan was performed; however, no extravasation was found due to backflow through the cephalic artery.

The following day, the huge hematoma was removed under general anesthesia, and it was confirmed that there was no bleeding from other vessels. The patient was discharged from the hospital on day X+11. She underwent outpatient rehabilitation and recovered without any discomfort during hand movement.

## Discussion

According to the National Institutes of Health Consensus Development Conference Statement, NF1 is diagnosed if two or more of the seven characteristic features are present [5]. This diagnosis does not include vascular fragility, making it difficult to diagnose the first vascular problem. Vascular fragility associated with NF1 has been reported in a few cases, with an incidence ranging from 2.3% to 3.6% [1,2]. Vascular fragility cannot be visually determined due to the mixture of normal and abnormal vessels. Vascular fragility lesions have been reported in the renal artery (41%), carotid, vertebral, and cerebral artery lesions (19%), and lesions in the extremities (< 4%) [6]. The rate of fragility in the vessels of the extremities is so low that diagnosing vasculopathy in undiagnosed NF1 cases as arising because of NF1 complications is very difficult.

Reubi classified vascular histology into intimal, aneurysmal, and fusocellular forms [7]. Regarding etiology, Schwann cell proliferation within vessel walls is considered the major etiology in large vessels, whereas mesodermal dysplasia or fibromuscular hyperplasia mainly occurs in small vessels [8,9].

In Gustavo’s study, aortic fragility was localized from the aorta to the renal artery, suggesting that the spread of NF1 vasculopathy to nearby vessels may be intermittent [6]. This is in accordance with the idea that the etiology of vascular abnormalities in large and small vessels is different. With accumulating case reports and the ability to differentiate abnormal vessels, large-vessel type, small-vessel type, or mixed-type vascular fragility can be distinguished in the future.

Severe bleeding due to the vulnerability of the inferior vena cava during nephrectomy following TAE for ruptured renal aneurysms has been reported [3]. This case report indicates that vascular vulnerability extends not only to the arteries but also to nearby veins. Bargiela et al. reported that in 66 endovascular cases, the rates of major and minor complications were 15% and 6%, respectively, with four cases (6%) of perioperative deaths [4]. However, there are challenging cases, such as that reported by Higa et al., where patients underwent repeated endovascular surgery three times due to pseudoaneurysms in intra-abdominal vessels [10]. Difficulties in endovascular treatment (EVT) may differ between the trunk and extremity vessels.

In the indications for open surgery or endovascular approach, Matsuura et al. suggested that the endovascular approach is limited by anatomical problems and lesion accessibility. Further, they suggest that because of these limitations, open surgery remains the treatment of choice in some cases [11]. In a report by Takata et al. using a combination of stent graft and open surgery for a ruptured celiac artery, reoperation was performed twice for SMA and bleeding in the right hepatic artery [12]. Kobayashi et al. reported the recurrence of a pseudoaneurysm in the postoperative repair of a radial artery aneurysm, and the patient underwent reoperation to ligate the radial artery [13]. Considering these reports, open surgery in the current situation, where vascular fragility cannot be grossly confirmed, may cause damage to the surrounding vessels. Endovascular treatment has limitations in terms of anatomical problems and lesion accessibility, whereas open surgery may lead to complications of postoperative bleeding owing to invasion of the surrounding vessels. Therefore, under the current circumstances, the superiority of endovascular treatment over open surgery cannot be confirmed.

In our case, extravascular leakage occurred during manipulation near the insertion site of the brachial artery, possibly due to vascular vulnerability extending from the rupture site to the proximal vessels. This suggests that the assumption that a proximal puncture site is safer may not be entirely accurate. However, this vulnerability may extend to the trunk vessels. For EVT of the extremities, puncture at the proximal portion, which is less likely to be a major complication, should be considered.

One month after discharge, the patient underwent a laparoscopic oophorectomy, during which no intraperitoneal vascular hemostasis or vascular fragility was noted. This suggests that the etiology of vascular fragility in small and large vessels is different, as discussed above. NF1 is an autosomal dominant gene and has no medical treatment. However, the risk of future bleeding from the vessels surrounding the fragility lesions remains. Therefore, patients need to be informed about the possibility of developing new symptoms.

## Conclusions

Vascular fragility is a rare complication of neurofibromatosis type 1. We reported a case of left ulnar artery rupture. In this case, we treated endovascularly by puncturing the ipsilateral brachial artery, achieving excellent results. The number of patients with vascular fragility is small, and treatment strategies for vascular fragility of the trunk and extremities are not established. There may be differences in safety and difficulty between EVT of the trunk and extremities. There are frequent reports of serious complications associated with EVT or open surgery to the trunk vessels. Although the possibility of fragile proximal vessels should be considered, EVT by proximal vessel puncture is considered safer for the extremities.
